# Working from home for good? Lessons learned from the COVID-19 pandemic and what this means for the future of work

**DOI:** 10.1007/s11573-022-01124-6

**Published:** 2022-11-24

**Authors:** Christian Kagerl, Julia Starzetz

**Affiliations:** 1 Institute for Employment Research (IAB), Regensburger Strasse 104, 90478 Nuremberg, Germany; 2 Faculty of Business and Economics, University of Basel, Peter Merian-Weg 6, 4002 Basel, Switzerland

**Keywords:** Working from home, Remote work, Coronavirus, COVID-19, Firms, D22, J24, M21

## Abstract

In the wake of the COVID-19 pandemic, more firms than ever before have enabled their employees to work from home. Based on a representative firm survey in Germany, surveying 2.000 firms per month throughout the course of the pandemic (October 2020 until June 2022), this paper provides suggestive evidence concerning the effects of working from home (WFH) at different points in time during the pandemic and discusses implications for the future of work. We assess the potential of WFH in Germany to be 25–30% of private-sector employees. On the firm side, we find that higher WFH use is positively related to business success during the crisis, with increased employee productivity and employees working more hours when remote being possible mechanisms. Larger firms in particular are open towards expanding their WFH offerings in the future. During the pandemic, firms have experienced that WFH has worked well in many respects (e.g., productivity of employees, quality of work performed) and, for the future, they are willing to facilitate WFH in order to give their employees more flexibility, and to be considered an attractive employer. However, working on site brings advantages (e.g., communication, cooperation and onboarding of new employees) firms will not want to sacrifice, pointing towards a hybrid model of work.

## Introduction

Beginning with the lockdowns in March 2020, the COVID-19 pandemic has forced firms to move much of their work from corporate offices to their employees’ homes. Within a few days, firms were required to revise entire work processes and to replace, where possible, the otherwise common face-to-face contact with digital alternatives. The effects of this shift in the way of working are of interest to researchers and practitioners alike, and, with this paper, we contribute by presenting early findings on the role of WFH during the pandemic in Germany and discussing its implications for the future of work. Based on data available to us, we consider the following questions: Who is able to WFH? What experiences have firms had with WFH during the lockdowns? How have firms which enabled WFH weathered the crisis and was WFH use related to business success? Is WFH establishing itself as a new, relevant form of work which is thus becoming a central component of a new mode of working after the pandemic has subsided?

In this paper, we are focusing in particular on the firms’ point of view and thus contribute by bringing a new perspective to an area of research which has predominantly focused on employees so far (e.g., Baumann and Kohlrausch 2021, Barrero et al. 2021a, Emanuel and Harrington 2021, Etheridge et al. 2020, Sahni 2020, Yang et al. 2021). Instead of looking at consequences on an individual employee level (e.g., on motivation, well-being, work-family conflict), we look at the experiences firms have had with WFH during the COVID-19 pandemic and underlying productivity changes. We consider this perspective to be of particular interest as the operational consequences of WFH are likely to be decisive in determining whether WFH will continue to be facilitated by firms in the future. For example, if employers were to observe that employees use their increased autonomy to shirk, they would probably bring their employees back on site despite potential positive effects of WFH on wellbeing. Another argument in favor of analyzing the firms’ perspective is that the firms’ self-assessments regarding plans for the future of WFH can be considered very informative since these reflect the current sentiment of decision-makers and thus allow initial conclusions to be drawn about what can be expected in terms of WFH after the pandemic.

Compared to other studies which are also considering the firm perspective (e.g., Bartik et al. 2020; Erdsiek 2021), we differ in that we use comprehensive survey data that are representative of the German economy as a whole and which allow an analysis over time, being based on surveys conducted at different times during the pandemic. It is interesting to study Germany because of its average pre-pandemic prevalence of telework compared to EU member states (Milasi et al. 2020) and because, also in terms of digitalization, it is not known as a pioneer but is rather perceived to be among the moderate or lagging countries (Ambrosio et al. 2020). The fact that there was still room for improvement before the COVID-19 pandemic in terms of both WFH usage and digitalization makes it an interesting case, in addition to Germany being the largest economy in Europe. However, when studying Germany, it is important to keep in mind the country-specific economic structure, for example the importance of high-quality manufacturing (Thelen 2019).

To the best of our knowledge, there is no other study to date that provides a thorough overview of WFH in Germany during the COVID-19 pandemic, in terms of both temporal variation as well as thematic diversity. Originally, there was some uncertainty about how to work with the challenges of social distancing and whether WFH would be able to replace working on site. With the extensive survey results available to us and after more than two years of living with the virus, we are able to take a look back at the lessons learned (so far). At the outset, it was open whether, based on the experiences gained, we would all work from home for good after the pandemic or whether any WFH efforts would be discontinued. In the remainder of this paper, we will use the term “working from home” (WFH) to refer to a concept that describes the work of an employee in his or her private sphere and that, unlike teleworking, does not necessarily require a fixed workstation (Backhaus et al. 2021). This term best describes the reality that could be observed during the pandemic, since due to the rapid progression of events in early 2020, hardly any conventional workstations could be set up at the employees’ homes, but most employees have created a more or less improvised workplace for themselves.

We find that, with the first COVID-19 infections in the beginning of 2020, there was a sharp increase in firms enabling WFH which reached a constantly high level of almost 50% during the course of the crisis. Calculations regarding the WFH potential, i.e., the proportion of employees who can potentially work from home, show a share of 25–30% of private-sector employees which was almost completely exhausted, especially during lockdown periods in early 2021. Moreover, we find suggestive evidence that WFH firms fared better during the pandemic. Among mechanisms explaining this observation are productivity increases when WFH and employees working more hours at home. Regarding a post-COVID-19 future, the majority of larger firms (with more than 250 employees) plans to expand their WFH offering as compared to the time before the crisis. Overall, WFH seems to work well in many firms (e.g., the assessments of work ethic/team spirit, of productivity of employees, and of quality of work are net positive) although there are also areas which confronted firms with difficulties (e.g., communication, on-boarding of new employees). In particular for smaller firms that plan to continue remote work, working exclusively or predominantly remotely seems to be an option while larger firms are more likely to plan a hybrid way of working that, in addition to WFH options for up to two days per week, includes regular office attendance.

This paper is structured as follows: Sect. 2 gives an overview of the current state of research. In this context, we present findings from before and the most important early results from during the COVID-19 pandemic, also providing a timeline of events in Germany that were relevant for WFH during the crisis. Then, we turn to the establishment survey,^1^ describe the data set (Sect. 3), and outline our key results in three steps. First, we present general firm-level results regarding WFH before and during the COVID-19 pandemic (Sect. 4). Second, we investigate the impact of WFH on establishments during the COVID-19 pandemic, also considering possible mechanisms (Sect. 5). Third, in Sect. 6, we discuss firms’ plans for WFH after the pandemic has subsided. Section 7 concludes.

## Literature review

With the spread of the first information and communication technologies (ICT), the idea emerged that employees could perform tasks from outside their offices. For the first time in the 1970s, in order to reduce commuting times, employees in the information industry in the US were enabled to do their work close to or from their homes (Nilles 1975). Since then with the emergence of smaller and lighter devices, work has increasingly become independent of place and time. While more and more employees are being given the opportunity to work from home, it is not yet completely resolved how WFH affects employees’ performance and thus a firm’s overall results. Basically, the question is whether employees use the autonomy they gain through WFH to shirk, since they can no longer be monitored so well, or whether their intrinsic motivation stays or even increases as a result with a positive impact on employee effort. While supporters particularly emphasize the positive effects on a better work-life balance, employee satisfaction, and thus employee motivation, which are supposed to go hand in hand with a better performance, others doubt whether these positive effects are present at all and claim that employees use the greater autonomy to shirk. Yahoo, HP and IBM are well-known examples of companies in which the management has restricted or even completely abolished the possibility of WFH, which had existed for years (Cirigliano and Niemeyer 2020). In these cases, the reasons given were a lower willingness to perform on the part of WFH employees and a lack of communication and interaction possibilities within and between organizational units (e.g., Green et al. 2020). This corresponds to the often-invoked lack of “water-cooler conversations” when WFH that supposedly leads to declining employee creativity and less innovation.^2^ Overall, for most companies, letting employees work from home implies decentralization of decision-making, which can be perceived as a strong loss of control (Aghion et al. 2014).

The difficulty in fully encompassing the effects of WFH lies, among other things, in the complexity of the measure which is very closely related to many different aspects of employees’ professional and private lives (e.g., work-family conflict, job satisfaction, health outcomes, career perspectives, interpersonal cooperation, innovation) the effect of which is often, also from a theoretical perspective, open (Allen et al. 2015). In addition, there are disagreements about the definition of WFH, about how it should be measured and there are few experimental settings which allow meaningful results (Bloom et al. 2015; Dutcher 2012). However, in these cases, mainly positive effects of WFH on productivity can be found. Moreover, Rupietta and Beckmann (2018) find that the more is worked from home, the more pronounced the effect on employee effort.

With the advent of the COVID-19 pandemic and the measures taken to contain it, irrespective of the previous opinions about WFH, suddenly more or less every firm had to deal with the option to let employees WFH where tasks made that feasible. According to the International Labor Organization (2020), globally, more than 15% of jobs could be done from home. In developed countries (like Germany or the US), this proportion is estimated to be around 25% by the ILO. Based on a job classification conducted by Dingel and Neiman (2020), the projected share is even higher, at 37% in the US. Accordingly, jobs that can be performed from home are primarily well-paid jobs^3^ which also explains why countries with a lower income level have a lower WFH potential.^4^ These disparities related to countries, income, and education are also highlighted by other studies which is why already existing inequalities between countries may increase further in pandemic times (Adams-Prassl et al. 2020, 2022; Bartik et al. 2020; Barrero et al. 2021b; Bick et al. 2020; Bonacini et al. 2020; Gottlieb et al. 2020; Hatayama et al. 2020). Furthermore, there are also differences in the industries and hierarchy levels in which WFH is possible or more common. Here, again, it is often the case that more highly qualified, often non-physical employees (e.g., knowledge-intensive work, business service providers) are more likely to be able to WFH and were laid off less frequently during the current crisis period than if they have had a lower level of education and did physical work (e.g., work in capital intensive industries, hospitality, leisure) (Bartik et al. 2020; Brynjolfsson et al. 2020).

In the current literature on WFH during the COVID-19 pandemic, some studies have addressed the effects that the rapid increase in WFH has had on both employees and employers, although much of the literature has focused on evidence from the employee side. Among the positive experiences employees have had while WFH in the last few months is an improved work-life balance and work efficiency as well as greater work control (e.g. Angelici and Profeta 2020; Ipsen et al. 2021). Negative effects were particularly evident in the form of declines in mental well-being, in difficulties to perform work when family obligations had to be fulfilled at the same time and in WFH not being a perfect substitute to office work due to, e.g., the lack of (technical) equipment and/or the limitations of virtual collaboration (Etheridge et al. 2020; Ipsen et al. 2021). When mentioning these disadvantages or negative effects of WFH, however, it is also important to point out the exceptional situation that prevailed during this period, for example, school closings and contact restrictions in general. In terms of productivity, the available studies found no or hardly any negative effects, rather, being able to work from home seemed to help mitigating the negative effects of the crisis (Alipour et al. 2021b; Angelici and Profeta 2020; Etheridge et al. 2020).

Regarding the future of WFH after the pandemic, based on an employee-level study, Barrero et al. (2021a) found evidence that there will be a strong shift towards more WFH which can be explained, at least in part, by a reduced stigma regarding WFH. Accordingly, negative expectations were often not confirmed. On the contrary, WFH worked better than expected in most cases. Consequently, these developments should have positive effects in the long term as should the fact that many places have invested in a technical infrastructure. For Germany, Bellmann et al. (2021) document an increase in investments in digital technologies in the wake of the pandemic, concentrated among larger employers and knowledge-intensive sectors. These investments are strongly tied to changes in WFH with firms particularly spending on hardware, communication software (e.g., Zoom, Microsoft Teams) and remote access capabilities. Based on the changes made during the pandemic, Barrero et al. (2021a) further predict that productivity will increase as soon as the crisis have been overcome due to the increase in WFH. Erdsiek (2021), who presents the results of an employer survey which is representative for the German information and manufacturing industries, also expects more work to be done from home even after the pandemic. Similar to the study by Barrero et al. (2021a), he justifies this by the employers’ positive experiences made with WFH, for example, barely any negative productivity effects could be observed, and by the technical progress and the far-reaching investments made in this very short time frame.

**Fig. 1:**
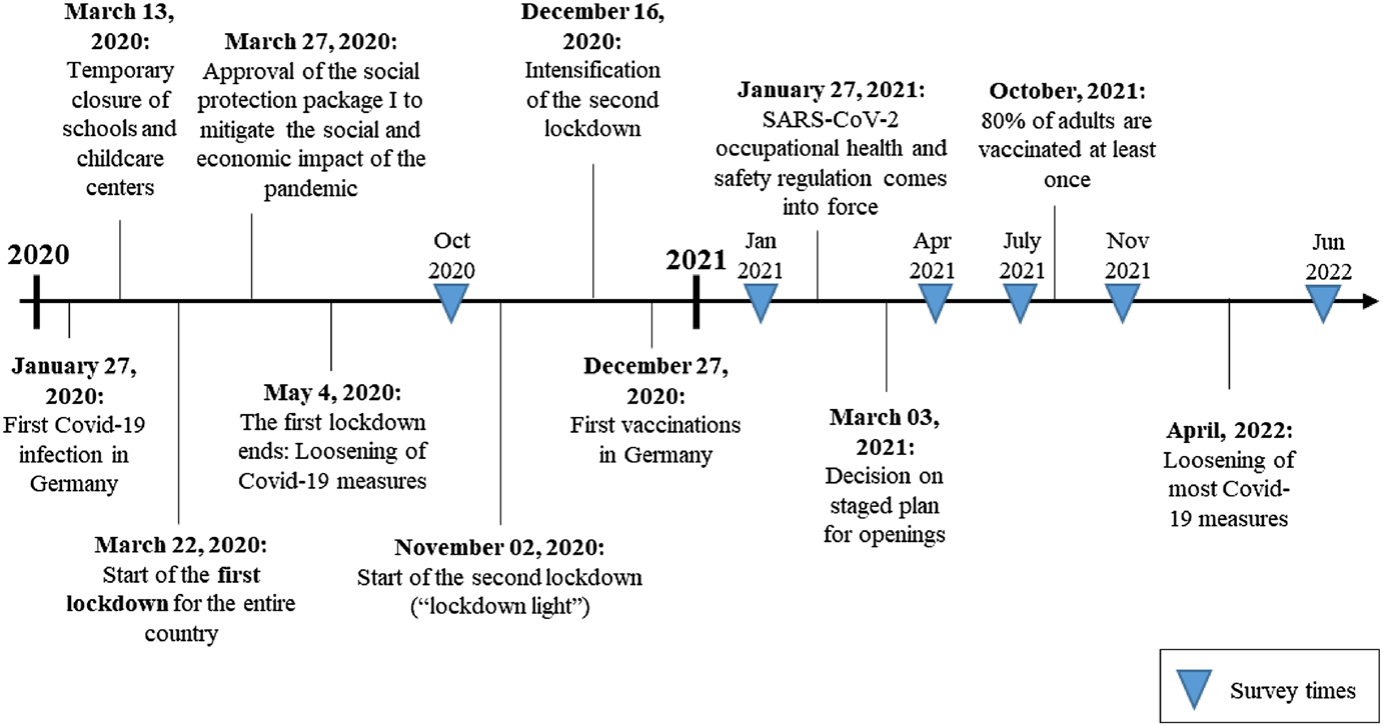
Chronology of the COVID-19 Pandemic in Germany. Source: Own graphical illustration based on reports from the German Federal Ministry of Health (2021), Handelsblatt GmbH (2021), Deutsche Welle (2021)

Figure 1 displays important events and decisions with regard to our research question during the COVID-19 pandemic in Germany as well as the times when surveys of the Institute for Employment Research with questions about WFH were conducted. Germany was for the most part in lockdown between March and May 2020 and between November 2020 and March 2021. During these periods, public life was kept to a minimum and citizens were instructed to socially distance themselves to the greatest possible extent. Many establishments suffered greatly from these restrictions and were helped in the form of social protection packages to mitigate the social and economic impact of the pandemic. During the second lockdown, SARS-CoV-2 occupational health and safety regulation came into force. In this context, distance and hygiene measures were defined and employers had to allow WFH wherever feasible. Finally, in April 2022, most of the COVID-19 measures were loosened again, including the obligation to wear a mask in many areas. With regard to the chronology of the COVID-19 pandemic in Germany displayed in Fig. 1, the question arises as to what effects could be observed over the course of time with respect to WFH. Based on the data set introduced in the next section and approaching the topic from the (so far understudied) firms’ perspective, we examine whether the literature’s findings discussed so far can be confirmed, what specific experiences have been made in Germany and whether there have been changes throughout the pandemic’s phases.

## Data

We analyze data from an establishment survey that was repeatedly conducted by the Institute for Employment Research in Germany during the COVID-19 pandemic (Backhaus et al. 2022; Bellmann et al. 2022). Starting in August 2020, roughly every month 1.500–2.000 establishments were surveyed on the varied effects of the crisis and how firms have adapted to it. Due to the high frequency of survey waves, the study is designed as a rotating panel, i.e. after a couple of participations, establishments were rotated out of the sample in order to ease the burden and keep response rates stable. The samples are drawn from the establishment file of the Federal Employment Agency, which contains all privately owned establishments in Germany that are required to provide notification within the social security system. As the vast majority of establishments are small, a simple random sample would be unlikely to include enough large establishments which, however, have a considerable share of total employment. Hence, a disproportionate sampling scheme was applied where establishments with more than 250 employees were oversampled to ensure that, in each survey wave, there is a sufficient number of these.^5^ Concretely, the goal of at least 150 large establishments (with more than 250 workers) was met in each survey wave. After the completion of each wave, a weighting scheme was applied to make the given (roughly 2.000) establishments in that particular survey wave representative with respect to the private sector in Germany. The weighting followed three main steps: First, using the survey design, inclusion probabilities were computed based on the establishment file. Second, these weights were updated based on further field, contact and administrative data to adjust for potential selection due to unit non-response.^6^ Third, the weights were calibrated using generalized regression. This third step calibrates the weights in such a way that the sum of all weights in a survey wave matches the total number of establishments in the strata, while–simultaneously–the sum of all weights, where each weight is multiplied with the establishment’s employment size, also matches the total employment in the strata. Hence, two sets of weights can be applied. The first set of weights, establishment weights, makes each sample representative for the establishment population. The second set, employment (or: activity) weights, makes each sample representative for overall employment in the private sector in Germany, i.e., it allows us to gauge what WFH means in terms of worker shares (overall and for those able to WFH), not just in terms of establishment shares.

As the survey was initiated to broadly capture the impact of the pandemic, WFH was not part of each survey wave. The questionnaires covered, on the one hand, a set of recurring questions such as on the direct effects of the pandemic and the measures to contain it, and, on the other hand, a flexible module which has changed in the respective survey waves. Within these modules, the topic of WFH was covered multiple times, first in October 2020 and then at various points during 2021 (also see Fig. 1). Some questions on WFH were repeated in each WFH-wave, e.g., which firms made WFH possible, an estimate for the WFH potential, and the number of employees currently having the option to work from home. Further questions focused on how firms see WFH affecting their operations, how they assess the future of WFH, and which reasons are pertinent in establishments wanting to (not) increase the possibilities of WFH. Table 3 in the Appendix gives a broad overview over some of the WFH topics that were surveyed and when. In general, the survey questions were answered by managerial staff of the establishment, although this differed by establishment size. Typically, in smaller establishments, the owner responded to the survey, whereas in larger establishments the type of respondent shifted, often to the head of the human resources department.

While, due to the survey structure, establishments were surveyed multiple times, the panel structure is, unfortunately, of very limited use in our case as most waves with questions on WFH were months apart and the overlap leaves a too small number of establishments for informative analyses. However, we could link the surveyed responses with administrative data on the establishments from before the onset of the pandemic coming from the Establishment History Panel (BHP).^7^ The key advantage of these administrative records is that they allow us to observe some establishment characteristics that are not available from surveys, e.g. median wages within firms and the employment trend for the years prior to the crisis. The information on the workforce composition is particularly relevant for the topic of WFH. We observe the shares of employees by gender, by nationality, and–crucially–by occupational requirement levels (similar to skill levels) as well as by broad occupational groups.

## WFH before and during the COVID-19 pandemic

How much was worked from home before and at different points in time during the pandemic? Which kind of firms have made WFH possible? What are the barriers to WFH and why are firms reluctant to implement WFH? This first step is intended to give an overview of what has happened in the months of the pandemic in Germany with regard to WFH and on how profound changes were in comparison to before COVID-19 hit the country.

### WFH over the course of time

**Fig. 2:**
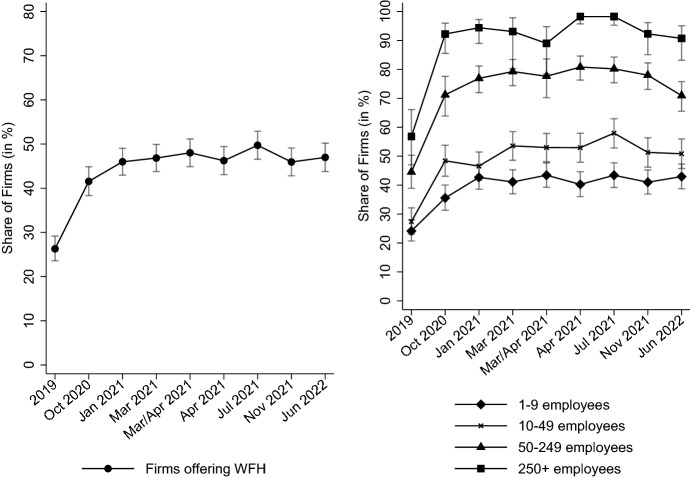
WFH over time in establishments and by establishment size. Establishment shares are weighted to be representative of all private-sector establishments in Germany. 95%-CIs are shown. The information on WFH before the pandemic was asked retrospectively in 2021. Since firm exit regulation changed during the pandemic, fewer firms were exiting than before, making it very unlikely that the values for 2019 exhibit a survivor bias

First, we consider the extensive margin of WFH, i.e., which establishments implemented WFH. Implementation usually entails that a firm has rules in place regarding the usage of WFH, which we take to be a definition of WFH that focuses on employees having the option to work full days remotely. Figure 2 shows the share of establishments that enabled WFH for (at least a part of) their workforce between 2019 and 2022 (left panel). Whereas before the pandemic, 25% of establishments enabled WFH, nearly half did make it possible in the summer of 2021. In contrast to the other points in time in Fig. 2, the information on the extensive margin of WFH in 2019 was asked retrospectively during the pandemic. Therefore, it might be possible that the number is inflated by recall bias. Also, it needs to be noted that Fig. 2 only shows changes in the extensive margin of WFH, therefore understating the shift towards WFH in the pandemic because increases in the intensive margin of WFH were happening as well (see Sect. 4.3 below and, in particular, Fig. 4). Still, many establishments have had their first ever experience with WFH when the pandemic started as 40% of establishments that resorted to WFH in the crisis have not made it possible at all for their workforce before the pandemic hit. While the share of establishments enabling WFH was roughly 50% in 2021, that half of firms accounts for 75% of total employment in Germany, hinting at strong differences by firm size. Yet, as we show below in Sect. 4.3 covering the intensive margin of remote work, WFH is often only possible for a subset of an establishment’s workforce.

In the right panel of Fig. 2, differences in establishment size are displayed. WFH was made possible in many large firms from very early on in the pandemic and this level has remained stable at over 90% since. Smaller establishments–which constitute around three quarters of the approximately 2.1 million establishments in Germany–switched less often to WFH initially, but an increase could be recorded over the course of the crisis, from 36% in October 2020 to 43% in July 2021.

The relevance of establishment size for enabling WFH is also borne out by regressing the binary indicator of WFH possibility on a broad range of variables to determine what correlates with the adoption of WFH. As shown in Table 4 (in the Appendix), a regression pooled across the survey waves yields that establishments with 250 or more employees have, on average throughout the crisis, a probability of enabling WFH that is 40 percentage points higher than for small establishments with less than ten employees, conditional on holding different firm attributes and the workforce composition fixed. Apart from the strong gradient in establishment size, some other variables have robust associations with WFH. Among others, establishments in the ICT sector as well as firms active in the service industry are more likely to offer WFH relative to production firms. Both export activity and higher log median wages correlate strongly positively with the possibility of WFH. Further, there is a positive time effect with WFH being significantly more likely to be offered in the first half of 2021 compared to October 2020. Also, as one would expect from the literature discussed above, the workforce composition plays a prominent role. More specifically, here, the firms’ shares of requirement levels from 2019 were used as a proxy for the occupational structure. Requirement levels describe what level of skills are demanded within occupations. These are closely linked to education^8^, e.g., the majority of Germans works in specialist occupations (the omitted category in Table 4), which require having completed vocational training. Similarly, highly complex occupations usually necessitate a university degree. Tasks, on average, become more cognitively oriented, more abstract and more analytical with an increasing requirement level, therefore making those occupations more amenable to WFH. Indeed, estimates from Table 4 suggest that a share of highly complex occupations that is 10 percentage points larger, ceteris paribus robustly increases the probability of offering WFH by about 6 percentage points.

### Barriers to WFH

Although there has been a major increase in WFH use in the pandemic as compared to before the pandemic, there is still a fairly large proportion of firms that do not offer WFH at all, despite the general situation during the pandemic requiring that as many employees as possible actually work from home in order to minimize the general risk of infection. This leads to the question of the barriers that make WFH infeasible, or the barriers because of which employers are reluctant to introduce WFH options. In the fourth survey wave, in October 2020, establishments which did not offer WFH were surveyed on the reasons for this decision. The majority of firm that did not offer WFH stated that this was due to a lack of suitability of the tasks being performed within their firm (98%). Other reasons, more widespread among small establishments, were the lack of technical equipment such as mobile devices and access to firm servers (37%) as well as that data protection and data security regulations generally did not permit it or would make the implementation of WFH prohibitively expensive (25%).

While activities that are not suitable for WFH are probably difficult to change in the short term, the proportion of WFH establishments could presumably be increased through targeted adjustments with regard to the two other barriers mentioned. In Sect. 5, we will discuss the role of investments in technical infrastructure in more detail. Concerning data protection, it is important to note that cybersecurity threats, which were already assessed as a considerable risk prior to the pandemic, have now taken a new dimension as the vulnerabilities firms faced in these times of crisis have increased the opportunities for cyber criminals to do harm (Ahmad 2020; Pranggono and Arabo 2021; Williams et al. 2020). Accordingly, firms that offered WFH during the pandemic were increasingly facing problems due to phishing and ransomware attacks which specifically exploited the concerns and difficulties of individuals in setting up a workspace at their homes by themselves. However, in general (also irrespective of WFH), the pandemic has exposed many (cyber) security gaps which, on the one hand, explain the firms’ concerns in terms of allowing WFH but, on the other hand, reveal the general need for the revision of firms’ security concepts, although cyber defense may require costly investments in technical infrastructure as well as in employee training.

### WFH potential

Even though about three quarters of all employees in Germany work in firms which enabled WFH at least to some degree during the pandemic, not all occupations and tasks performed in these establishments were also done (or doable) from home. For example, a large plant might have sent some administrative staff to WFH, but out of necessity retained physical presence for production workers.

**Fig. 3:**
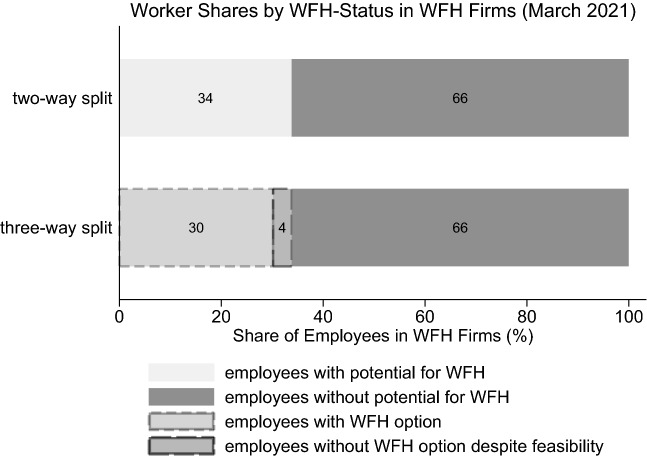
Types of WFH Shares. Shares are employee-weighted

Within the survey, employers offering WFH were asked about how many employees they deem able to WFH in theory based on to their task structure (we call this the “WFH potential”) and, subsequently, how many were actually given the option to WFH.^9^ Specifically, we split an establishment’s workforce into various subgroups: First, there is the share of employees that could WFH (“employees with potential for WFH”) which is defined as the number of potential remote workers as judged by the establishment divided by the total number of employees. The second subgroup (“employees without potential for WFH”), defined as the proportion of employees who cannot work from home due to their tasks, by construction adds up to 100% with the first subgroup; all employees can therefore be mapped into these two subgroups. However, the first group can be split up once more by how many employees were actually given the opportunity to work remotely. This results in, on the one hand, the share of employees that was given the option to WFH and, on the other hand, the share of workers with jobs feasible for WFH but without the opportunity to do so. This last group can be interpreted as the unused potential of WFH. On average, across WFH firms, about half of the workers are classified as being able to WFH in theory. Yet, weighting by overall employment suggests that two thirds of all employees across WFH establishments do jobs unsuitable for WFH. Using employment weighted proportions and March 2021 as an example, Fig. 3 exemplifies how the workforce in all WFH establishments can be split up into the aforementioned groups.

**Fig. 4:**
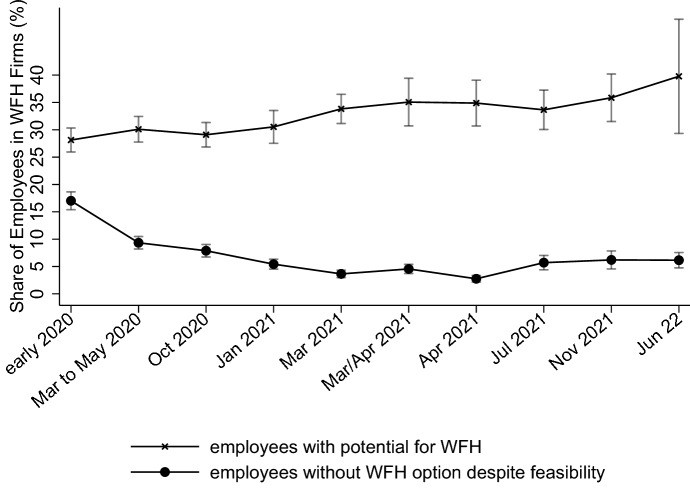
(Unused) WFH potential in WFH firms over time. Shares are employee-weighted. 95%-CIs are shown. The information on WFH right before the pandemic was asked retrospectively in October 2020. Since firm exit regulation changed during the pandemic, fewer firms were exiting than before, making it very unlikely that the values for early 2020 exhibit a survivor bias

Figure 4, for all firms that offer WFH, then plots how two of these proportions, the WFH potential and the unused WFH potential, have developed over the course of the pandemic. The WFH potential has increased slightly, but not significantly so, likely tied to the fact that short-time work prevented many layoffs in Germany. However, establishments extended the intensive margin of WFH as a growing number of their employees with suitable tasks were given the possibility to work remotely. While, before the pandemic, more than half of the potential went unused, this share has almost steadily dropped until spring 2021, before significantly increasing again afterwards.

Given the data at hand, no causal explanation can be discerned, but there are multiple potential factors that could explain this development. The trends in the WFH potential, for example, line up with temporary regulations aimed at containing the pandemic that were put into place by the German Federal Government between January and June 2021. The SARS-CoV-2 Occupational Health and Safety Regulation, which was in force in this time period, included measures for reducing the likelihood of infection while at work, e.g., through mandating that employers offer antigen tests. In 2021, from January until the end of June, establishments were also required to make WFH possible for all employees unless the establishment had explicit reasons against WFH. While the unused WFH potential declined after the regulation took effect and increased again after it ran out, this could also be due to other reasons. For one, the virus was surging in winter and ebbed going into spring and summer along with vaccinations becoming available to all adults, potentially leading firms to adjust their WFH offers. However, county case numbers of COVID-19 show no association with the WFH possibility in the pooled sample and there is no change in the usage of the potential in November 2021 despite a strongly increasing number of infections during that month. Furthermore, the WFH possibility could also depend on employee demands unrelated to federal regulations. In general, though, most workers in Germany with the opportunity to work from home had access to it during the pandemic, and continue to have access in 2022. Although no direct evidence is available to us on employees’ take-up of WFH possibilities before the pandemic, it stands to reason that many more workers actually used their WFH offer or, in some instances, were even forced into WFH by their employers. Roughly 20% of establishments (mostly larger ones) had at one point until November 2021 obligated their employers to WFH and recommendations for using WFH were given in over 60% of firms.

The results also allow to calculate the share of workers (excluding the public sector as it is not part of the survey) in Germany who have the potential to WFH. Multiplying the approximately 75% of all workers accounted for by WFH firms with the roughly 33% of feasible jobs within those firms yields a WFH potential of about 25% of the workforce. Prior results for Germany vary widely − 29% are estimated by Boeri et al. (2020), 37% is the number by Dingel and Neiman (2020) and 56% according to Alipour et al. (2021a)–placing our estimate at the lower end of the spectrum. Importantly, most measures rely on job characteristics or on worker-level evidence, e.g. from surveys on tasks, while our number uses estimates from employers. Therefore, it is likely that establishments resort to a narrower definition of who can work from home, for example by judging employees to not be among the potential WFH group if a small share of their tasks could be done from home since it would possibly not make much sense to have these workers WFH from the business’s perspective. Similarly, employers, in contrast to employees, might only consider those to have potential to work from home which can work full-length days remotely. Such a conceptual difference between *theoretical* and *practical* WFH capacity could explain the wide discrepancies in the estimated potentials. Further, our estimate should be interpreted as a lower bound since there might be untapped potential among the establishments never offering WFH (which we would miss), although that is likely to be small with many of such firms clustering in sectors like hospitality or construction. Still, to provide some insight on the potential scale of the underestimation, we take the potential share of WFH as the dependent variable in a (fractional response) logit model, regressing it on a rich set of survey and administrative variables,^10^ pooling the survey waves. Specifically, we only consider the first appearance of a firm in the survey, exclude those establishments that do not offer WFH at all (for which we have no information on the potential) and use the estimated coefficients to predict the potential WFH share for all establishments, including those never making WFH possible. Using the predictions from this exercise in combination with the employment weights suggests an economy-wide WFH potential of 30%. Because the independent variables cannot fully capture differences in the potential between WFH and non-WFH establishments, the estimation relying only on WFH firms makes the resulting proportion very likely an upper bound. Therefore, overall, the firm survey results suggest that there is a WFH potential between 25 and 30% in the private sector in Germany.

## WFH and business success

Apart from reducing contacts and thereby aiming to slow the spread of SARS-CoV-2, what were the experiences and effects of increased WFH use on firms? Was WFH an extra burden during a severe economic crisis or a useful tool for successfully weathering it? What are mechanisms through which any effects on business success manifest themselves? At first, motivated by findings that WFH can increase employee productivity (Barrero et al. 2021a; Bloom et al. 2015), we examine if and how the use of WFH is related to various measures of business success during the pandemic. We run regressions of the following type to establish first suggestive evidence:

1 $$Y_{f} = \alpha + \beta *WFHshare_{f} + \gamma *X^{\prime}_{f} + \varepsilon_{f}$$

When investigating the connection between WFH and business success, our main independent variable of interest is the share of employees the respective firm ***f*** has given the opportunity to work from home (*WFHshare*). ***Y*** refers to the respective outcome variable measuring business success. In this Section, we have several outcome categories (*Crisis Effects, Revenue Change, Profitability*), constructed from the survey as follows:

- *Crisis effects:* A binary variable equal to one if the firm reports that the COVID-19 crisis has negative economic effects on the establishment. Available in all waves.
- *Revenue change:* How revenue developed in the month preceding the interview compared to that same month the year before–[Available for three waves; we compare the category “decreasing revenue” to the categories “increasing revenue” and “revenue staying roughly the same”].
- *Profitability:* A dummy whether the firm has turned a profit in 2020, only available for firms surveyed in July 2021.

On the right-hand side of Eq. (1), the vector ***X*** captures a rich set of control variables, partially motivated by the correlating factors of WFH adoption shown in Sect. 4. More concretely, the controls can be split into the following subsets:

- *Establishment characteristics:* These encompass, first, the sector of the establishment as well as its size which both are strongly correlated with the option of WFH. Further characteristics concern the existence of a works council, whether the establishment is foreign-owned, whether it is part of a larger company, its age and its region type (urban vs. rural).
- *Workforce characteristics:* Most importantly, these characteristics taken from administrative data include worker shares by occupational requirement level (akin to the skill level), a determinant of what type of tasks are done. Moreover, the median wage and the mean age within the establishment are also part of this subset of controls, as are worker shares by gender, foreign and minor employed.
- *Pre-pandemic characteristics:* These variables capture the aspect that WFH firms and non-WFH firms might already systematically differ before the pandemic initially hit, i.e., were more productive or had a different time trend. As a measure of pre-pandemic productivity, we use the establishment’s position in the AKM-type fixed effects (Abowd et al. 1999) distribution calculated from the period 2010–2017 (Bellmann et al. 2020). We also incorporate the establishment’s overall employment trend between 2016 and 2019.

**Fig. 5:**
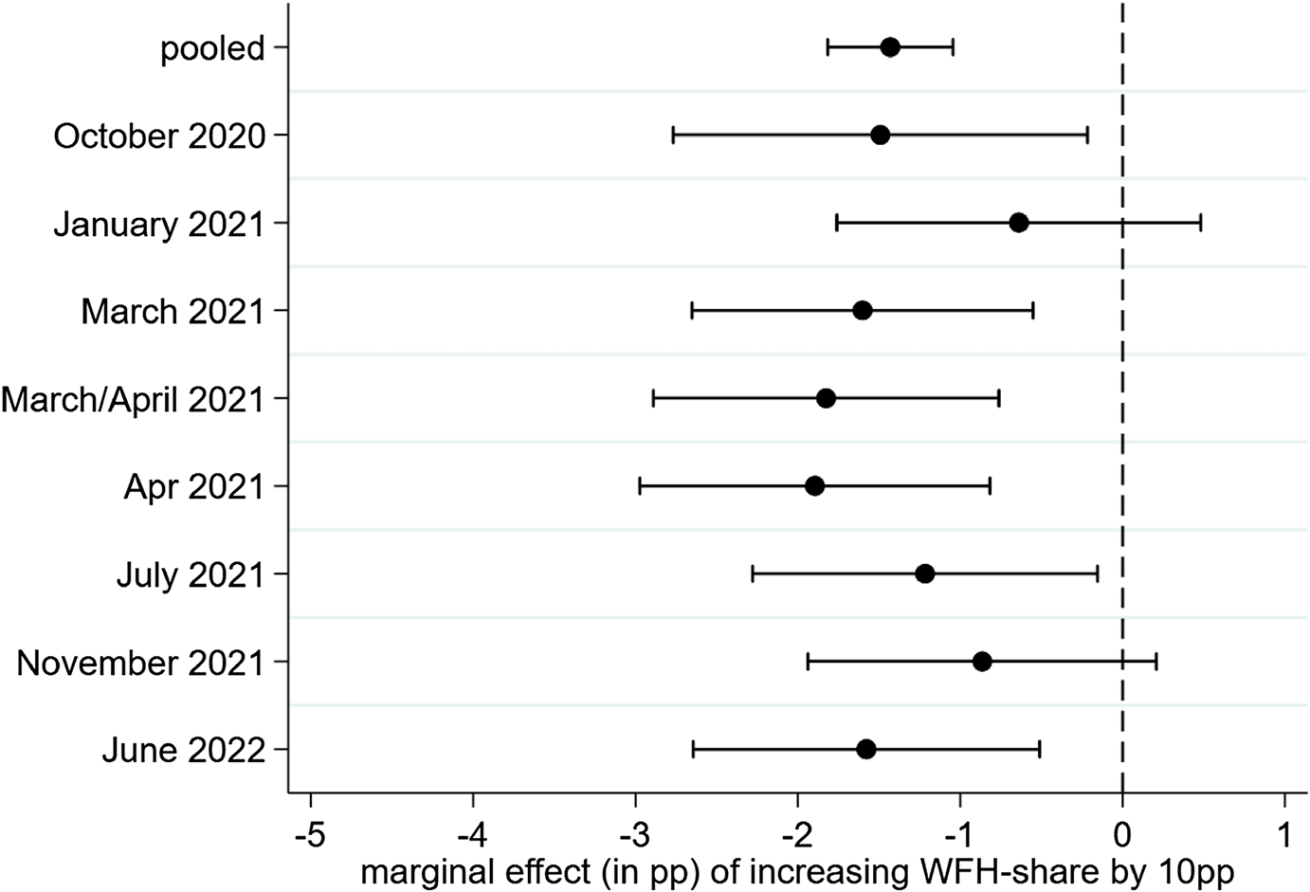
WFH and Crisis Effects. 95%-CIs are depicted. Average marginal effects from logit regressions, standard errors clustered on the establishment level. The pooled specification also includes a set of indicators for the survey waves

Returning to Eq. (1), ${\varvec{\beta}}$ yields the association between WFH and the business success outcome variable conditional on all the control variables. ${\varvec{\alpha}}$ is a constant and ${\varvec{\varepsilon}}$ represents the standard errors, which we cluster on the establishment level. Using crisis effects as the dependent variable, Fig. 5 shows results for $\beta$ obtained from implementing Eq. (1), once pooled across all survey waves (further including wave indicators) and then separately for each point in time. Estimates are relatively stable and suggest that increasing the WFH share by about ten percentage points ceteris paribus is associated with a decrease in the probability of reporting negative consequences due to the pandemic by 1.5–2.5 percentage points.

This pattern is very similar if we consider whether firms’ revenue has decreased compared to the previous year. An increase in the WFH share by 10 percentage points is associated with a two percentage points lower probability of decreased revenue (see Fig. 12 in the Appendix). Additionally, WFH also correlates with profitability. Running specification (1) with profitability as the dependent variable (survey wave from July 2021; further controlling for pre-crisis profitability in 2019) reveals that increasing the WFH-share by 10 percentage points increases the probability of turning a profit in 2020 by 1.3 percentage points (S.E. of 0.4). Taken together, we thus find suggestive evidence that higher WFH use is positively related to business success outcomes during the crisis. In a next step, we are now interested in finding reasons for why this might be the case.

### Mechanisms

Considering a simple production function, WFH has in principle two levers through which it can affect firm outcomes. First, WFH could impact labor productivity, i.e., the output produced by a given unit of labor input changes. Here, prior evidence from the employee side suggests that WFH increases productivity (Barrero et al. 2021a; Bloom et al. 2015). Second, WFH could change the amount of labor input (even when holding employment levels fixed), i.e., employees change how many hours they work. According to Rupietta and Beckmann (2018), employees’ work effort increases when WFH. Also, evidence from worker surveys during the pandemic indicates that employees work more when WFH, a result of time saved on commuting being partially reallocated to work (Barrero et al. 2021a). Further, both channels do not exclude each other and could hence happen simultaneously. We first discuss these two channels in the context of our firm survey and then investigate the mechanisms between WFH and business success with the data at hand.

#### The productivity channel

In November 2021 and June 2022, establishments were asked how they see WFH impacting their employees’ productivity. 22% of firms report an increase in productivity due to WFH, 13% a decrease and 60% no consequences (the remaining 5% fall into the category “don’t know”). When weighting by the employment population that is able to work from home, 27% of employees able to WFH are considered to be more productive when WFH and 9% to have a lower productivity relative to working in the office (also see Fig. 10 in Sect. 6.2). Interestingly, none of our standard control variables in *X* seem to have meaningful predictive power about the productivity assessments of WFH.

**Fig. 6:**
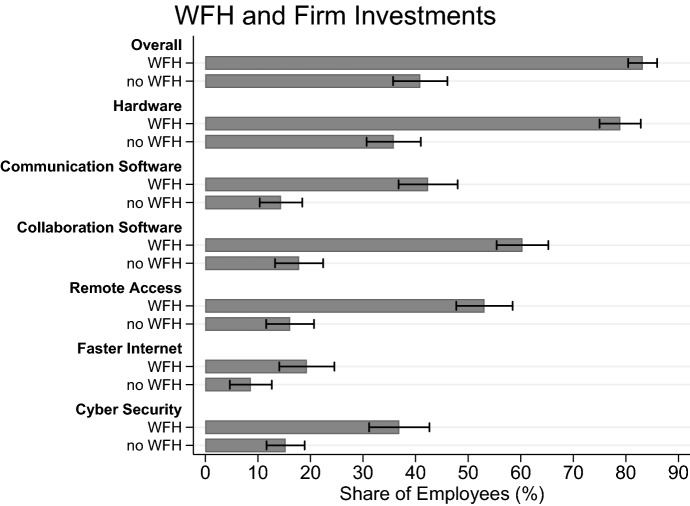
Investments in Digital Technologies and WFH. 95%-CIs are depicted. Shares are weighted to be representative of all private-sector employees in Germany. The information on investments comes from February 2021, whereas the data on WFH come from the same establishments at different points in time (either January, March or April). N = 1.506 establishments

Further, it is important to keep in mind that productivity changes when WFH can have a variety of causes. For one, productivity may have changed due to the characteristics of the new working environment (e.g., due to a reduction in distractions). Another reason could be that new labor-complementary technologies, which have experienced a big push during the pandemic (Bellmann et al. 2021), have had an impact on productivity. In February 2021, firms were asked about their investments in IT and digitalization since the start of the pandemic. While two thirds of establishments that offer WFH invested in digitalization efforts during the first year of COVID-19, only one third of those not offering WFH did so. Figure 6, weighted by employment, shows sizeable differences in investments by the WFH-status of the firm. Among employees in firms with WFH, more than 80% saw their employer invest in at least one type of digital technology compared to 40% of employees in non-WFH firms.^11^

In terms of different areas of technologies, employees in WFH firms are much more likely to come into contact with and profit from digitalization across all types of digital technologies which are important or complementary to effective WFH, e.g. hardware, software for collaboration and communication, remote access capabilities as well as cyber security. Having the right digital tools can be conducive for productive WFH and can further facilitate employees acquiring skills from which they may benefit later. Correspondingly, the provision of IT-related training during the pandemic is much more widespread in WFH firms (35%) than in firms without a WFH option (8%).

Unfortunately, the sample of establishments that answered the questions on investments is different from the one that answered the questions on the effects of WFH on various aspects (among them worker productivity). Therefore, we assume that the more informative variable about the reported effect of WFH on productivity captures the potential productivity mechanism, although we do not know the exact source of the productivity change. In essence, we construct a variable (*ProdIncr*) that equals one if a firm reported that WFH increased the productivity of its employees, and zero otherwise.

#### The hours worked channel

As part of the questionnaire in June 2022, firms were asked about how they perceived employees’ working time to be affected when working remotely. The vast majority (about 80%) of establishments saw no difference. Roughly 15% reported an increase in hours worked and only a small portion (5%) specified that their employees were working less when at home. These proportions are very similar when weighted by employment (15 and 3%). This suggests that firms observe little to no shirking when employees work from home and thus confirms findings from employee-level studies (e.g., Bloom et al. 2015; Rupietta and Beckmann 2018). Analogously to the first channel, we construct a binary variable (*HoursIncr*) that is equal to one if a positive impact on time spent working is reported, and zero otherwise.

#### Specification

Our main independent variable of interest continues to be the share of employees the respective firm *f* has given the opportunity to work from home (*WFHshare*). To test the two channels of productivity (*ProdIncr*) and working time (*HoursIncr*), we interact the WFH-share with each of the two aforementioned dummies. Since we only have information on the effect of WFH on productivity and hours worked for one survey wave (from June 2022), we rely on the following cross-sectional specification to provide evidence on the mechanisms:

2 $$\begin{aligned} Y_{f} & = \alpha + \beta *WFHshare_{f} + \psi *ProdIncr_{f} + \tau *\left( {WFHshare_{f} \times ProdIncr_{f} } \right) \\ & \quad + \,\,\xi *HoursIncr_{f} + \theta *\left( {WFHshare_{f} \times HoursIncr_{f} } \right) + \gamma *X^{\prime}_{f} + \varepsilon_{f} \\ \end{aligned}$$

***Y*** refers to the respective outcome variable measuring business success. For this particular survey wave, we have two outcomes, *Profitability* and *Negative Crisis Effects*:

- *Profitability*: Same variable as above, but for the year 2021.
- *Negative crisis effects*: Same binary indicator as above.

Again, ***X*** captures the set of control variables as specified above, with the addition of controlling for profitability before the pandemic in 2019 as well. ${\varvec{\beta}}$ yields the association between WFH and the respective outcome variable conditional on all the control variables. Correspondingly, ${\varvec{\beta}} + {\varvec{\tau}}$ gives the marginal effect of the WFH-share if a firm reports WFH influencing productivity positively, ${\varvec{\beta}} + {\varvec{\theta}}$ the marginal effect if WFH has led to employees working more and ${\varvec{\beta}} + {\varvec{\tau}} + {\varvec{\theta}}$ if both mechanisms apply. This exercise therefore tries to tease out which channel correlates with business success by comparing the obtained marginal effects. We, again, cluster standard errors on the establishment level.

Table 1 shows results with *Profitability* as the dependent variable, introducing the interaction terms to the estimation column by column. Without any interaction terms present in the estimation, in the first column, there is a positive (but weak and insignificant) relationship between the WFH share and profitability. Columns 2 and 3, separately, include the two interactions with *ProdIncr* and *HoursIncr.* Results suggest that there is a significant connection to *Profitability* if WFH is considered to increase worker productivity or hours worked. In terms of magnitude, the coefficient in the second column implies that each increase in the WFH share by 10 percentage points leads to a 1.4 percentage points higher probability of turning a profit in 2021. However, this only applies if the firm reports a positive impact of WFH on productivity, which suggests that *ProdIncr* is a possible channel. The coefficient in the third column for *HoursIncr* can be interpreted equivalently. Finally, in the fourth column both interaction terms are included simultaneously in a “horse race” regression, i.e. the full specification as given by Eq. (2). The relationship is strongest when both mechanisms apply. Yet, for *Profitability*, the estimates indicate that – on its own – only the *HoursIncr* channel is significantly associated with turning a profit during the crisis and it therefore seems, rather than *ProdIncr*, to be the driving force.

**Table 1:** WFH and Profitability (Mechanisms)

Interactions included	Dependent variable: profitability in 2021			
	None	ProdIncr	HoursIncr	Both
	(1)	(2)	(3)	(4)
ME of WFH-share $\left( {\varvec{\beta}} \right)$	0.045 (0.047)	0.027 (0.051)	0.025 (0.051)	0.021 (0.053)
ME of WFH-share if ProdIncr > 0 $\left( {{\varvec{\beta}} + {\varvec{\tau}}} \right)$		0.143* (0.086)		0.050 (0.102)
ME of WFH-share if HoursIncr > 0 $\left( {{\varvec{\beta}} + {\varvec{\theta}}} \right)$			0.198** (0.092)	0.181* (0.107)
ME of WFH-share if ProdIncr > 0 and HoursIncr > 0 $\left( {{\varvec{\beta}} + {\varvec{\tau}} + {\varvec{\theta}}} \right)$				0.209** (0.102)
Controls	Yes	Yes	Yes	Yes
Establishments (N)	1527	1527	1527	1527

Standard errors (in parentheses) clustered at the establishment level. ME abbreviates marginal effect. Stars denote significance levels: * = 10%, ** = 5%, *** = 1%. Establishments surveyed in June 2022

In Table 2, we replicate the approach from Table 1, but change the dependent variable to the *Negative Crisis Effects* outcome. Using this measure, we find that the WFH share is connected to a lower probability of reporting negative crisis ramifications (first column, also see Fig. 5). Again, the connection is more pronounced when positive effects of WFH on worker productivity or working time are observed by the firm (columns (2) and (3)). The “horse race” specification in column (4) indicates that enabling a 10 percentage points higher share of the workforce WFH reduces the probability of being negatively affected by the crisis by 2.4 percentage points, if both mechanisms apply. On their own, the productivity channel seems to be more relevant here, although the estimates are relatively imprecise.

**Table 2:** WFH and Negative Crisis Effects (Mechanisms)

Interactions included	Dependent variable: self-reported negative effects of pandemic in 2022			
	None	ProdIncr	HoursIncr	Both
	(1)	(2)	(3)	(4)
ME of WFH-share $\left( {\varvec{\beta}} \right)$	− 0.126** (0.051)	− 0.092* (0.054)	− 0.097* (0.054)	− 0.084 (0.055)
ME of WFH-share if ProdIncr > 0 $\left( {{\varvec{\beta}} + {\varvec{\tau}}} \right)$		− 0.229** (0.097)		− 0.207 (0.126)
ME of WFH-Share if HoursIncr > 0 $\left( {{\varvec{\beta}} + {\varvec{\theta}}} \right)$			− 0.195* (0.100)	− 0.119 (0.132)
ME of WFH-share if ProdIncr > 0 and HoursIncr > 0 $\left( {{\varvec{\beta}} + {\varvec{\tau}} + {\varvec{\theta}}} \right)$				− 0.242** (0.108)
Controls	Yes	Yes	Yes	Yes
Establishments (N)	1507	1507	1507	1507

Standard errors (in parentheses) clustered at the establishment level. ME abbreviates marginal effect. Stars denote significance levels: * = 10%, ** = 5%, *** = 1%. Establishments surveyed in June 2022

Regarding the question which WFH channel correlates more strongly with business success, our results are not conclusive. Taking *Profitability* as an outcome, the horse race regression (Table 1, column (4)) suggests that the association between WFH and profitability during the pandemic is driven by employees working more rather than worker productivity gains per se. The same approach with *Negative Crisis Effects* as the dependent variable (Table 2, column (4)) suggests a larger role for productivity changes over working time changes, although not significantly so. Hence, both mechanisms might be relevant for explaining the link between WFH and business success. One possibility why the data here remain inconclusive is that there are substantial heterogeneities in how WFH might affect productivity and working time between workers, even within firms. For instance, productivity differences between departments can be observed, such as productivity increases for call center employees (as found by Bloom et al. 2015), but only minor productivity gains for workers from engineering, marketing and finance (Bloom et al. 2022). Since in our survey, there is only one overall response for each firm in which all divisions are included, these heterogeneities cannot be disentangled with the data at hand.

Finally, we would like to point out one further possible mechanism that could play an important role in the context of our research question, namely the association between the pandemic shock and the ability to WFH. Specifically, firms with tasks amenable to WFH were less likely to be disrupted by the pandemic and its countermeasures, precisely because the strictest measures were placed on businesses where staff and customers directly interact. Hence, the shares of occupational requirement levels (see above) are important to adjust for, as are the industry controls since the latter were used for delineating the stringency of government measures. Nevertheless, our rich set of controls might not fully capture this differential affectedness. Correspondingly, we do not interpret our evidence as strictly causal.

## WFH in the future

With a possible link between WFH and business success during the pandemic, what will the future hold for WFH? Is WFH establishing itself as a new, relevant form of work which is thus becoming a central component of a “new normal” in firms, or are firms scaling back to the WFH level prior to the COVID-19 pandemic? Based on the results in Sect. 5, we would expect WFH to stay an integral part of the world of work moving forward. Because establishments were queried with regard to their planning for after the pandemic as part of the surveys, we can draw some (preliminary) conclusions for the future of work.

### WFH for good?

**Fig. 7:**
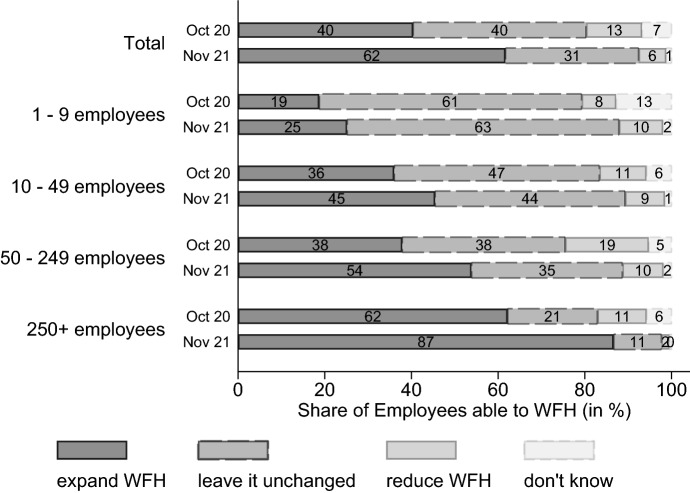
To what extent do you intend to give your employees the option of working from home or teleworking in future, when the current pandemic is over? Compared to the time before the crisis, do you intend in future to. Shares are weighted to be representative of all employees able to WFH in private-sector establishments in Germany. N_oct20_ = 1.052 establishments and N_nov21_ = 1.178 establishments

Twice during the pandemic, once in October 2020, just before the start of a long winter lockdown, and one year later, in November 2021, with again increasing COVID-19 case numbers, establishments were asked how they plan to proceed with WFH in the future in comparison to the pre-pandemic WFH situation. Figure 7 plots the results. In October 2020, 40% of employees who were able to work from home due to their tasks, worked in a firm which could envision an expansion of WFH in the future. By November 2021 that share had risen to 62%. The share of WFH-able employees working in firms wanting to reduce WFH decreased (from 13 to 6%), as did the share in establishments aiming for pre-pandemic levels of WFH (from 40 to 31%). Looking at the breakdown by establishment size, it becomes apparent that the larger the establishment, the greater the willingness to offer more WFH after the crisis. Small firms with fewer than 10 employees appear to be mostly satisfied with their pre-pandemic work arrangements, while for a majority of workers in large firms (with more than 250 employees), employers are considering to expand WFH. This large proportion which is planning to expand WFH suggests that experiences with WFH during the crisis were mostly positive, in particular in larger firms. Moreover, for all establishment sizes, there is the same time trend of firms adjusting their plans towards more future WFH. Between the two survey waves, firms had one additional year of experience with WFH which they incorporated into their plans and which appeared to lead them to increase the role of WFH. Additionally, plans have gotten more concrete as the share of employees in firms not knowing about future WFH yet decreased from 7 to 1%.

**Fig. 8:**
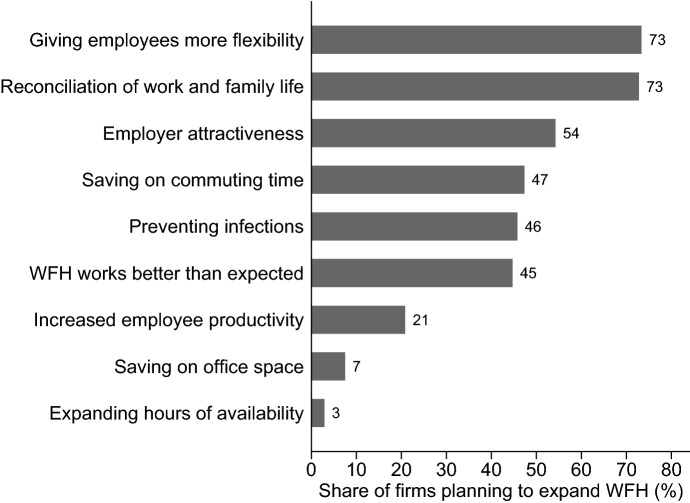
Why are you aiming to expand the use of working from home or teleworking?. Establishments could give answers on a scale from one to five, with five being “applies entirely”, represented constitute those answering four or five. Shares are weighted to be representative of all private-sector establishments in Germany. Question was asked in October 2020, N = 272 establishments

What are the main reasons behind the firms’ plans? In October 2020, establishments that wanted to expand WFH and those that planned to reduce WFH or leave it unchanged in the future as compared to the time before the crisis, have been asked follow-up questions regarding the reasons for their plans. Among firms planning to expand WFH in the future (Fig. 8), the most frequently cited reasons for this decision were, first, to permit a better reconciliation of work and family life and, second, to give their employees more flexibility (73% each). Furthermore, employers seem to anticipate that many employees have come to appreciate benefits of WFH during the pandemic and that it will therefore be important in the future to offer WFH options in order to being considered an attractive employer (54%). Furthermore, in a later survey wave in June 2022, more than half of WFH firms rated employee demands for continued remote work as important reason for sticking to WFH. Already at an early stage of the pandemic, a noticeable WFH boom could be observed in online job advertisements (Alipour et al. 2021b).

The finding by Barrero et al. (2021a) at the employee-level that a pre-existing stigma associated with WFH has diminished can be confirmed at the firm-level with the data set available to us: More than half of the firms that plan to expand WFH in the future reported that WFH worked better than expected. In contrast to the skepticism observed in various firms before the pandemic, one in five firms that want to expand WFH wants to do so in order to increase productivity. While 47% mention a reduction in commuting time as another reason, merely 7% do so for reducing office space, a topic that has received considerable attention in the popular media as a purported consequence of the change to WFH. Very few establishments state that they want to expand the hours of availability of their employees (3%).

**Fig. 9:**
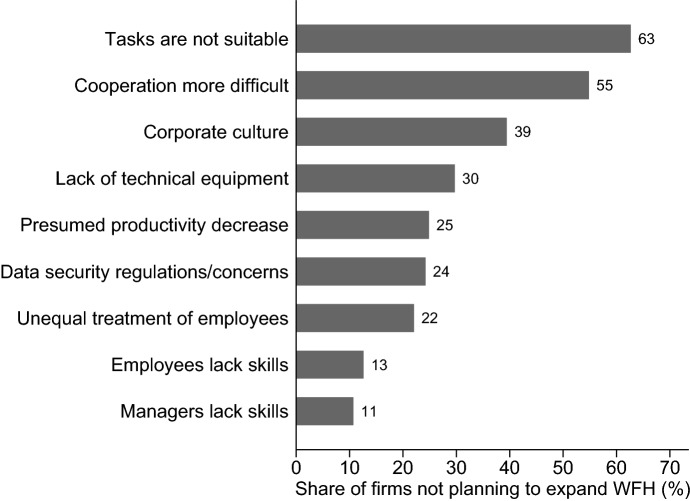
Why are you aiming to return to the same or a lower level of working from home as compared to before the COVID-19 crisis?. Shares are weighted to be representative of all private-sector establishments in Germany. Question was asked in October 2020, N = 702 establishments

Also, those firms that intended not to expand their WFH were asked about the reasons for their plans (see Fig. 9). The most frequently mentioned reason for not expanding WFH compared to the pre-pandemic state is that tasks are not really considered suitable for WFH (63%). Since only firm that enabled WFH during the pandemic were surveyed for this question, this means that they gave WFH a chance, but did not rate it as equivalent to the non-WFH option. The further answer options provide information about how this assessment was formed. Accordingly, cooperation was considered more difficult (55%), employees’ performance was expected to be lower when WFH (25%) and managerial as well as non-managerial staff were supposed to lack skills (11% and 13%). As in the original question, which explored the reasons for not offering WFH, lack of technical equipment (30%) and data protection and data security regulations (24%) were also reasons for not expanding WFH, albeit to a reduced extent here. Furthermore, more than one in three firms surveyed said that they do not want to expand WFH as they want to avoid unequal treatment of employees. 39% of firms surveyed indicate that WFH does not fit their corporate culture and therefore they would like to leave their extent of WFH unchanged, or even reduce it as compared to the extent before the pandemic.

A glance at the distribution of reasons mentioned for not expanding WFH in the future gives the impression that WFH confronts firms with interpersonal challenges in particular. Technical feasibility and general productivity losses seem to be less of a problem than the fact that cooperation per se is made more difficult and people might feel unfairly treated with potential negative effects on corporate culture. Firms that discovered deficits with regard to technical equipment and/or their employees’ IT skills at the beginning of the crisis have already reacted in this respect. This hypothesis is supported by findings of Barrero et al. (2021a), Bellmann et al. (2021) and Erdsiek (2021) as well as by the results in Sect. 5 on what kind of investments have taken place (see Fig. 6 above). Approximately two-thirds of the WFH firms surveyed stated that they have invested in digitalization during the pandemic, mostly focusing on acquiring hardware such as laptops, tablet computers, webcams, and headsets, and on software such as video-conferencing software or project planning tools. In addition, despite difficulties of moving training activities to a virtual format, 35% of firms enabling WFH have conducted IT-related training for their employees. Through these investments, firms have lowered important hurdles that would have originally made WFH difficult. It can thus be assumed that many employees are now equipped with the infrastructure and skills to continue using WFH in the future. In general, results indicate that many firms have made a leap forward in the use of new workplace technologies and in the development of remote work within a very short period of time which otherwise would have probably taken several years. This catalyzing influence can, for example, be seen in the rapid advancement of technologies and tools that promote virtual collaboration (Bloom et al. 2021).

### Impact of WFH and implications for the future

**Fig. 10:**
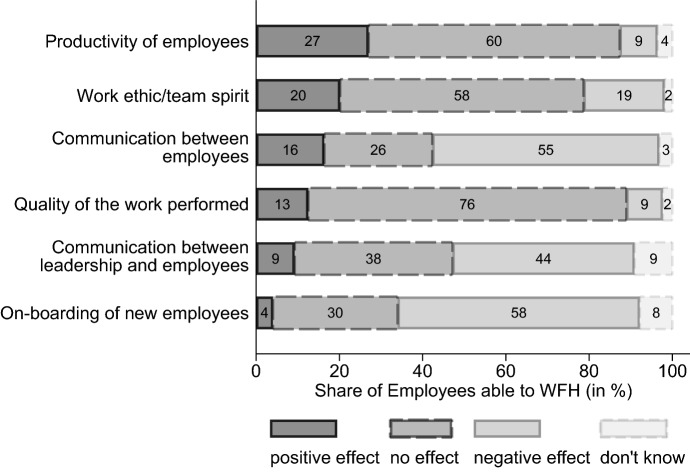
Assessment of the impact of WFH on different areas during the pandemic. Shares are weighted to be representative of all employees able to WFH in private-sector establishments in Germany. Establishment assessments from November 2021, N = 1.187 establishments

While the pandemic often necessitated experimenting with WFH, the question remains as to what lessons have been learned from this period and what a “new normal” might look like. In this context, firms’ assessments regarding the effect of WFH on different areas are revealing. Figure 10 shows areas where WFH works well and can produce positive outcomes, but also areas which are considered difficult or even problematic when working remotely. For 60% of employees who are able to work from home, according to their firms’ assessment, WFH did not affect their productivity. In addition, positive productivity effects are observed much more frequently than are negative ones (27 vs. 9%). Similarly, with regard to work ethic/team spirit and quality of work performed, positive effects were observed more frequently than negative effects. As laid out in Sect. 5, employees working remotely were also more likely to increase their hours worked than to decrease them. These assessments are in contrast to the shirking hypothesis widespread in corporate circles before the outbreak of the pandemic. Unlike previously expected, the WFH situation did not prevent employees from performing their work at a similar level of productivity and quality as before the pandemic, and also employee motivation did not seem to deteriorate in most cases as a result of the new work location. In total, this implies a positive effect of WFH on the overall economy if productivity increases for a sizeable subset of the workforce. This is further reinforced by the fact that firms that report negative productivity effects are much more likely to insist on using less WFH in the future.

Aspects that, however, worked less well when WFH from the perspective of many firms–particularly larger ones–were the communication both between employer and employees (44%) as well as among employees (55%), and the on-boarding of new employees (58%). During the pandemic, whenever WFH was possible, the usual face-to-face communication had to be largely abandoned and replaced by new mediums of communication which in some respects could only inadequately meet the needs of employers and employees, potentially leading to stress in organizations (Kirchner et al. 2021; Putri and Irwansyah 2020; Sahni 2020; Yang et al. 2021). For example, Yang et al (2021) find that there was a decline in synchronous communication which had a negative impact on information distribution and resulted in collaboration networks becoming more static. Probably also exacerbated by communication challenges, more than one in four establishments surveyed stated that WFH had a negative effect on the on-boarding process of new employees. Using the example of on-boarding of software developers, Rodeghero et al. (2021) find that not meeting your team in person has a negative impact on the social connection as well as on the integration into existing teams. This raises the question as to whether WFH during the pandemic may have worked well in part because the majority of teams already knew each other from before the crisis and had the opportunity to build relationships before which may have also been useful during the pandemic when WFH. Individual team members could still be integrated into existing team structures, albeit with difficulties, but how cooperation would work if an entire team has never met in person is uncertain and will probably have to be explored in future.

**Fig. 11:**
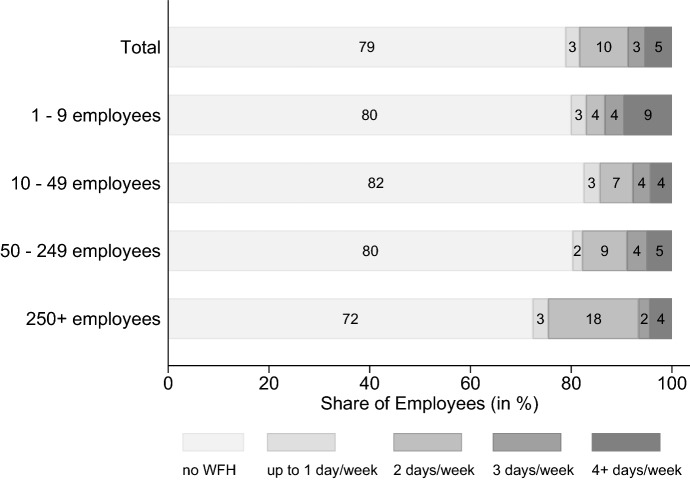
Planned future maximum WFH days per week for employees. Shares are weighted to be representative of all employees in private-sector establishments in Germany. Answers from November 2021, N = 1.097 establishments

**Fig. 12:**
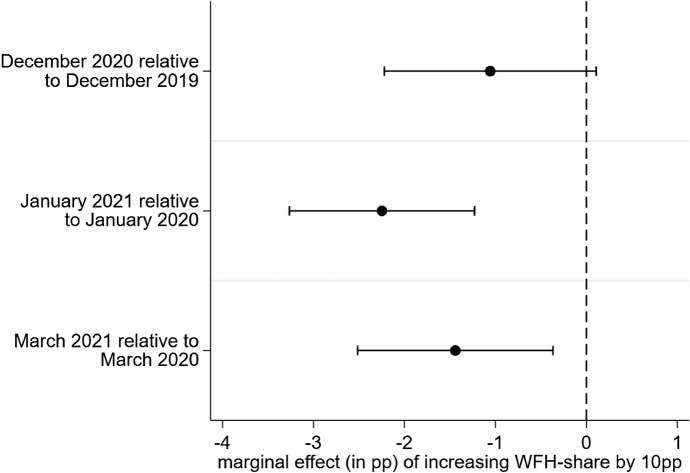
WFH and Revenue Changes. 95%-CIs are depicted. Average marginal effects from multinomial logit regressions, predicting “revenue decrease”. The other two categories are “revenue increase” and “revenue staying roughly the same”. Standard errors are clustered on the establishment level

Overall, while WFH has worked well, there are also areas which cannot be considered equivalent to their non-WFH counterpart. In the future, it will be possible to return to original workplaces and to implement new forms of work organization that both pick up advantages (e.g., productivity) and absorb disadvantages (e.g., social connections) of WFH. In this context, so called “hybrid solutions” seem possible which allow a combination of different work locations (Bloom 2021; Bloom et al. 2022). Within the survey, establishments were asked about the maximum number of days they would like to allow their employees to WFH in a post-pandemic future. Figure 11 shows how the firms’ plans for the future of WFH map to the employment population: 79% of employees will not be able to WFH, which aligns with the estimated WFH potential (outlined in Sect. 4.3) of 25–30%, indicating that most of this potential will be exhausted in the future as well. Of the remaining 21% being offered the opportunity to work from home, the majority will have the option to work remotely for a maximum of three days per week. With increasing firm size, the share of employees that can WFH for up to two days becomes larger. In establishments with more than 250 workers, nearly 20% of employees are projected to have this option. Hence, firms seem willing to embrace hybrid work for those employees with suitable tasks, especially among larger firms who want to keep some office attendance for coordination and socializing purposes. In this regard, it is further in the interest of the establishment to have fixed days for coming into the office. Additionally, Fig. 12 in the Appendix shows the planned future of WFH by sector. As expected, WFH will play a greater role in knowledge-intensive industries and in ICT.

## Conclusion

In this paper, we address the topic of WFH in different phases of the COVID-19 pandemic based on a unique establishment survey conducted by the Institute for Employment Research in Germany. The onset of the pandemic has led to a sharp increase in WFH, which was particularly evident in large firms (more than 250 employees), ICT firms and firm with a high proportion of highly complex occupations. The results, which are representative for the private sector in Germany, show a WFH potential of 25–30% which was almost completely exhausted by firms, especially in periods characterized by high case numbers. Relevant barriers preventing establishments to introduce WFH were a lack of suitability of the tasks being performed within their establishment, a lack of technical equipment as well as data security and data protection concerns.

Regarding the experiences made with WFH and the effects this changed way of working had on establishments, we find that WFH use is negatively related to reporting negative consequences due to the pandemic, but positively related to profitability. In terms of mechanisms explaining this crisis-mitigating role of WFH, we discuss and present evidence that gains in productivity and employees working more hours when WFH are channels that impact business success.

Finally, we consider how firm are planning their WFH for a time after the end of all pandemic restrictions. Overall, it is evident that many firms, especially larger ones, are planning to expand their WFH offering after the pandemic as compared to the time before the crisis. The most frequently mentioned reasons for these plans are to permit a better reconciliation of work and family life, to give their employees more flexibility, and to being considered an attractive employer. Among the reasons not to expand WFH were, next to the fact that jobs are not considered suitable for WFH, especially interpersonal challenges such as cooperation difficulties, non-suitability for corporate culture and fear of unequal treatment of employees. It appears that technical barriers have already been reduced in many cases through targeted investments in hardware and software as well as in IT training and IT support. While, according to the firms’ assessment, WFH has worked well in many respects (e.g., work ethic/team spirit, productivity of employees, quality of work performed), there are other factors which caused difficulties for the establishments that enabled WFH (e.g., communication, on-boarding of new employees). In light of these experiences, so-called “hybrid working arrangements” (Bloom et al. 2022) seem to be an option that exploit the advantages of WFH and mitigate its disadvantages. According to our results, in firms with more than 250 employees, roughly one fifth of all workers will have the option of WFH for up to two days per week. However, small firms (with fewer than 50 employees) that want to continue remote work can also imagine their employees WFH for a major part of the week (three or more days per week).

With our study, we provide a detailed overview of German firms’ experiences with WFH during the COVID-19 pandemic and its implications for the future of work. Our contribution here is in particular the focus on the firm perspective as well as the fact that we were able to compare different points in time during the pandemic with a great variety of topics addressed. Before the pandemic, WFH was more of a marginal phenomenon in Germany. Combined with a somewhat lacking digitalization, the first lockdown in March 2020 was a major challenge for many firms in Germany. At the beginning, there were many uncertainties as to how establishments would cope with this exceptional situation as well as whether and how this, for many, new form of working would work. For Germany, we could summarize first lessons learned from the pandemic and discussed how things could evolve based on descriptive results. An important finding that probably would not have been expected prior to the pandemic is that the majority of firms has had predominantly positive experiences with WFH (e.g., in terms of employee productivity, work ethic, and quality of work performed) and that 20% of the workforce in the German private-sector economy is projected to continue to use WFH in the future. Further, the transition to WFH was often accompanied and facilitated by investments into digital technologies.

Regarding the transferability of our findings to other countries, on the one hand, we would expect comparable experiences for firms in other, similar countries as the pandemic e.g., led to lockdowns in many countries. On the other hand, as something specific for Germany could be regarded, for example, the importance of the manufacturing sector, which may have become visible in the comparatively low estimated WFH potential, or the extensive social protection packages, through which negative economic effects could already be dampened independently of WFH. Overall, however, results from other countries (e.g., Barrero et al. 2021a, Dingel and Neimann 2020, Yang et al. 2021) give us some measure of confidence that the baseline findings apply to many countries regardless of these differences.

Closing this paper, it is important to note that, next to the descriptive survey evidence, we analyze conditional correlations which we do not claim to be causal. However, due to the scope of questions queried at different points in time and the fact that the dataset is considered representative of all privately owned establishments in Germany, we provide interesting early-stage evidence on the experiences with and ramifications of WFH from the firm perspective during the pandemic. Beyond this, it will certainly be interesting to see what the long-term effects on workplace autonomy will be and whether the firms’ plans for future remote work will change. Moreover, we assume that, as time goes on, the greater availability of data might allow informative comparisons between before, during, and after the pandemic, which will also improve the methodological possibilities for analysis, identification and thus, explanatory power of results. While our goal was to provide an overview of WFH during the COVID-19 pandemic in Germany from a business perspective, specific effects related to WFH mentioned in the course of the paper (e.g., collaboration, communication, motivation, mental health) may constitute fruitful avenues for further research.

## Figures and Tables

**Fig. 13:**
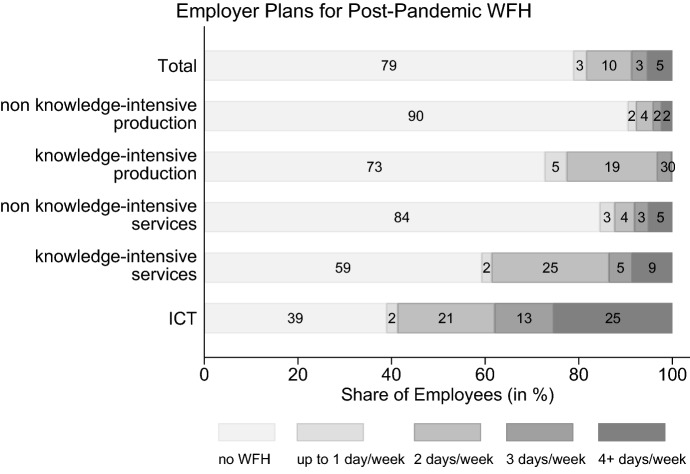
Planned future maximum WFH days per week for employees (by sector). Shares are weighted to be representative of all employees in private-sector establishments in Germany. Answers from November 2021, N = 1.097 establishments

## Data Availability

The data analyzed during the current study (“Establishments in the COVID-19 Crisis” (IAB BeCovid)) is available via on-site use or remote data access. Further information on data access can be found via: https://fdz.iab.de/en/betriebsdaten/panel-establishments-in-the-covid-19-crisis-iab-becovid-wave-01-24/ Nils Backhaus, Lutz Bellmann, Patrick Gleiser, Sophie Hensgen, Christian Kagerl, Theresa Koch, Corinna König, Eva Kleifgen, Moritz Kuhn, Ute Leber, Michael Moritz, Laura Pohlan, Swantje Robelski, Duncan Roth, Malte Schierholz, Sabine Sommer, Jens Stegmaier, Anita Tisch, Matthias Umkehrer, Armin Aminian (2022): Panel “Establishments in the COVID-19 Crisis”–20/21/22. A longitudinal study in German establishments–waves 1–24. FDZ Data Report, 09/2022 (en), Nürnberg. https://doi.org/10.5164/IAB.FDZD.2209.en.v1. The data access was provided via on-site use at the Research Data Centre (FDZ) of the German Federal Employment Agency (BA) at the Institute for Employment Research (IAB).

## Appendix

See Figs. 12 and 13, Tables 3 and 4.

**Table 3:** Questions on WFH During the Survey

Topic (broad)	October 2020	January 2021	March 2021	March/April 2021	April 2021	July 2021	November 2021	June 2022
Adoption of WFH	X	X	X	X	X	X	X	X
Potential for WFH	X	X	X	X	X	X	X	X
Effect of WFH: productivity							X	X
Effect of WFH: working time								X
Future of WFH	X					X	X	

X indicates that questions on the respective topic were included. However, questions need not have been identical. For example, establishments were asked about whether they wanted to expand WFH in the future in October 2020, July 2021 and November 2021. Yet, only in October 2020 there were questions on reasons for (not) expanding WFH–see Figs. 8, 9. And only in November 2021, there was a question on how many days WFH is planned to be possible in the future–see Fig. 11. More details on the questionnaire structure of the whole survey can be found in Backhaus et al. (2022)

**Table 4:** Correlates of WFH Adoption

Dependent variable = 1 if WFH is possible in establishment	(1)
	WFH (binary)
Sector, ref: non-knowledge intensive production	
Knowledge intensive production	0.0554*
	(0.0292)
Non-knowledge intensive services	0.0280**
	(0.0132)
Knowledge intensive services	0.189***
	(0.0175)
ICT (information and communic. tech.)	0.307***
	(0.0352)
Size, ref: 1–9 employees	
10–49 employees	0.109***
	(0.0141)
50–249 employees	0.306***
	(0.0157)
250 + employees	0.416***
	(0.0215)
Exporter	0.161^***^
	(0.0116)
Works council	0.0871***
	(0.0150)
Non-German ownership	0.0589**
	(0.0256)
Type, ref: single-plant establishment	
Headquarters	0.0807***
	(0.0150)
Branch	− 0.0288*
	(0.0156)
Age, ref: < 10 years	
> = 10 and < 25 years	− 0.0216
	(0.0136)
> 25 years	− 0.0253*
	(0.0136)
East Germany	− 0.0464***
	(0.0141)
Region, ref: urban Counties	
Semi-urban Counties	− 0.0518***
	(0.0128)
Semi-rural Counties	− 0.0669***
	(0.0148)
Rural Counties	− 0.0556***
	(0.0153)
Share unskilled/semi-skilled	− 0.0107
	(0.0220)
Share complex specialist	0.183***
	(0.0310)
Share highly complex	0.565***
	(0.0492)
Share German	0.102***
	(0.0293)
Share women	− 0.0863***
	(0.0180)
Share minor employed	− 0.0869***
	(0.0271)
Share in vocational training	− 0.0325
	(0.0564)
Mean age (in years)	0.00179**
	(0.000776)
Time, ref: October 2020	
January 2021	0.0237*
	(0.0134)
March 2021	0.0453***
	(0.0139)
March/April 2021	0.0536***
	(0.0141)
April 2021	0.0409***
	(0.0140)
July 2021	0.0712***
	(0.0141)
November 2021	0.0240*
	(0.0143)
N	12.411

Average marginal effects are shown. Standard errors (clustered on the establishment level) in parentheses. Significance levels: * = 10%, ** = 5%, *** = 1%. See text for more details

## Footnotes

1 Within the survey, the unit of observations are establishments (in German “Betriebe”). By this definition, an establishment is an independent organizational unit at a given location. Consequently, a firm could consist of several establishments. However, for the sake of simplicity, we will interchangeably refer to establishments as firms throughout the text.

2 For example, the CEO of JPMorgan, Jamie Dimon, stated in the Annual Report of 2020 (JPMorgan Chase & Co., 2020, p.46) that “[…] remote work virtually eliminates spontaneous learning and creativity because you don’t run into people at the coffee machine, talk with clients in unplanned scenarios, or travel to meet with customers and employees for feedback on your products and services”.

3 Dingel and Neimann (2020) find a positive correlation between the possibility to work from home and hourly earnings at the occupational level. Moreover, they point to a study by Mongey et al. (2020) who use the same occupational classification scheme, according to which not only do those who cannot work from home earn less, but they also have lower levels of education, rent more often than they own property and have limited healthcare.

4 In high-income countries many more jobs can be carried out from home than in low-income countries − 1/3 in contrast to 1/26 according to Garrote Sanchez et al. (2021).

5 The strata of the survey are defined through five broad economic sectors interacted with establishment size classes (in employees: 1–9, 10–49, 50–249, 250 +). Due to the oversampling, large establishments are taken to be one stratum, irrespective of their sector, leading to a total of 16 strata.

6 More details on the weighting can be found in Backhaus et al. (2022).

7 See Ganzer et al. (2020) for more details.

8 Throughout the paper, we focus on shares of requirement levels instead of education or schooling shares for multiple reasons. First, education variables in the BHP contain imputations but still leave a significant portion of persons with missing values. Second, the requirement levels come from the fifth digit of the occupational code and thus more closely capture the type of tasks being performed, e.g., eliminating potential mismatch. Third, as elaborated on in the main text, the requirement levels correspond to relevant qualifications in the German labor market.

9 Intermittently, the survey further asked how many employees were actually WFH, either fully or partially. But apart from fully remote situations, firms might not be able to accurately answer this when employees have flexibility regarding WFH. In order not to conflate employee reasons for not WFH (e.g., wanting to set boundaries between personal and work life) with employer reasons, we take the share (or number) of employees that have the possibility of WFH to be the variable of interest. Moreover, since we investigate establishment level outcomes, the offering of WFH more closely reflects the WFH decisions of firms.

10 Included are sector, size, age, exporter status, works council existence, German ownership, median wage, county type, mean age as well as employee shares by gender, nationality, minor employed, trainees, occupational requirement levels and nine broad occupational groups–also see Ganzer et al. (2020) for variable definitions.

11 This gap size also persists even when adjusting for a wide range of establishment and workforce characteristics.

## References

[CR1] Abowd J, Kramarz F, Margolis D (1999) High wage workers and high wage firms. Econometrica 67(2):251–333

[CR2] Adams-Prassl A, Boneva T, Golin M, Rauh C (2020) Inequality in the impact of the coronavirus shock: evidence from real time surveys. J Public Econ 189:104245

[CR3] Adams-Prassl A, Boneva T, Golin M, Rauh C (2022) Work that can be done from home: evidence on variation within and across occupations and industries. Labour Econ 74:102083

[CR4] Aghion P, Bloom N, Van Reenen J (2014) Incomplete contracts and the internal organization of firms. J Law, Econ Organ 30(1):i37–i63

[CR5] Ahmad T. (2020) Corona virus (COVID-19) pandemic and work from home: challenges of cybercrimes and cybersecurity. Available at SSRN 3568830

[CR6] Alipour JV, Falck O, Schüller S. (2021a) Germany’s capacity to work from home, CESifo Working Paper No. 8227

[CR7] Alipour JV, Langer C, O’Kane L (2021b) Is working from home here to stay? A look at 35 million job ads. Cesifo Forum 22(6):41–46

[CR8] Allen TD, Golden TD, Shockley KM (2015) How effective is telecommuting? Assessing the status of our scientific findings. Psychological Sci Public Interest 16(2):40–68 10.1177/1529100615593273 26403188

[CR9] Ambrosio F, Rückert D, Weiss C. (2020) Who is prepared for the new digital age. Evidence from the EIB Investment Survey, https://www.eib.org/attachments/efs/eibis_2019_report_on_digitalisation_en.pdf

[CR10] Angelici M, Profeta P. (2020) Smart-working: work flexibility without constraints. CESifo Working Paper No. 8165

[CR11] Backhaus N, Tisch A, Pohlan L, Kagerl C (2021) Working from home even after the coronavirus pandemic? Advantages and disadvantages from a business perspective. ASU Arbeitsmed Sozialmed Umweltmed 2021(56):276–284

[CR12] Backhaus N, Bellmann L, Gleiser P, Hensgen S, Kagerl C, Koch T, König C, Kleifgen E, Leber U, Kuhn M, Moritz M., Pohlan L, Robelski S, Roth D, Schierholz M, Sommer S, Stegmaier J, Tisch A, Umkehrer M, Aminian A. (2022) Panel “Establishments in the Covid-19 Crisis – 20/21/22”. FDZ Data Report 09/2022, https://doku.iab.de/fdz/reporte/2022/DR_09-22_EN.pdf

[CR13] Barrero JM, Bloom N, Davis SJ. (2021a) Why working from home will stick. National Bureau of Economic Research (NBER) Working Paper No. 28731

[CR14] Barrero JM, Bloom N, Davis SJ. (2021b) Internet Access and its Implications for Productivity, Inequality, and Resilience. National Bureau of Economic Research (NBER) Working Paper No. 29102

[CR15] Bartik AW, Cullen ZB, Glaeser EL, Luca M, Stanton CT. (2020) What jobs are being done at home during the COVID-19 crisis? Evidence from firm-level surveys. National Bureau of Economic Research (NBER) Working Paper No. 27422

[CR16] Baumann H, Kohlrausch B. (2021) Homeoffice: potenziale und Nutzung. Aktuelle Zahlen aus der HBS-Erwerbspersonenbefragung, Welle 1 bis 4. WSI Policy Brief No. 52

[CR17] Bellmann L, Gleiser P, Hensgen S, Kagerl C, Leber U, Roth D, Stegmaier J, Umkehrer M (2022) Establishments in the COVID-19-Crisis (BeCovid): a high-frequency establishment survey to monitor the impact of the COVID-19 pandemic. Jahrbücher Für Nationalökonomie Und Statistik 242(3):421–431

[CR18] Bellmann L, Lochner B, Seth S, Wolter S. (2020) AKM effects for german labor market data. FDZ Method Report 1/2020, https://doku.iab.de/fdz/reporte/2020/MR_01-20_EN.pdf

[CR19] Bellmann L, Bourgeon P, Gathmann C, Gleiser P, Kagerl C, Kleifgen E, König C, Leber U, Marguerit D, Martin L, Pohlan L, Roth D, Schierholz M, Stegmaier J, Aminian A. (2021) The pandemic has boosted firm investments in digital technologies. VoxEU 5 August 2021, https://voxeu.org/article/pandemic-has-boosted-firm-investments-digital-technologies

[CR20] Bick A, Blandin A, Mertens K. (2020) Work from home after the COVID-19 Outbreak. Federal Reserve Bank of Dallas Working Paper No. 2017

[CR21] Bloom N, Liang J, Roberts J, Ying ZJ (2015) Does working from home work? Evidence from a Chinese experiment. Q J Econ 130(1):165–218

[CR22] Bloom N, Davis SJ, Zhestkova Y (2021) Covid-19 shifted patent applications toward technologies that support working from home. AEA Papers Proceed 111:263–266

[CR23] Bloom N, Ruobing H, Liang J (2022) How hybrid working from home works out. National bureau of economic research (NBER) Working Paper No. 30292

[CR24] Bloom N. (2021) Hybrid is the future of work. Stanford institute for economic policy research (SIEPR): Policy Brief, June 2021

[CR25] Boeri T, Caiumi A, Paccagnella M (2020) Mitigating the work-safety trade-off. Covid Economics: Vetted Real-Time Papers 2:60–66

[CR26] Bonacini L, Gallo G, Scicchitano S (2020) Working from home and income inequality: risks of a ‘new normal’ with COVID-19. J Popul Econ 34(1):303–360 32952308 10.1007/s00148-020-00800-7 PMC7486597

[CR27] Brynjolfsson E, Horton JJ, Ozimek A, Rock D, Sharma G, TuYe, HY. (2020) COVID-19 and remote work: an early look at US data. National Bureau of Economic Research (NBER) Working Paper No. 27344

[CR28] Cirigliano L, Niemeyer J (2020) Zwiespalt Homeoffice. Eine Publikationsreihe des Schweizerischen Gewerkschaftsbundes, No, Dossier, p 143

[CR29] Dingel JI, Neiman B (2020) How many jobs can be done at home? J Public Econ 189:104235 32834177 10.1016/j.jpubeco.2020.104235 PMC7346841

[CR30] Dutcher EG (2012) The effects of telecommuting on productivity: an experimental examination the role of dull and creative tasks. J Econ Beha Org 84(1):355–363

[CR31] Emmanuel N, Harrington E. (2021) “Working” remotely? selection, treatment, and the market provision of remote work. Working Paper, 9

[CR32] Erdsiek D. (2021) Working from home during COVID-19 and beyond: survey evidence from employers. ZEW-Centre for European Economic Research Discussion Paper No. 21-051

[CR33] Etheridge B, Tang L, Wang Y (2020) Worker productivity during lockdown and working from home: Evidence from self-reports. Covid Economics 52:118–151

[CR34] Ganzer A, Schmidtlein L, Stegmaier J, Wolter S. (2020) Establishment history panel 1975–2019. FDZ Data Report 16/2020, http://doku.iab.de/fdz/reporte/2020/DR_16-20_v2_EN.pdf

[CR35] Garrote Sanchez D, Gomez Parra N, Ozden C, Rijkers B, Viollaz M, Winkler H (2021) Who on Earth can work from home? World Bank Res Observer 36(1):67–100

[CR36] German Federal Ministry of Health (2021). Coronavirus-Pandemie (SARS-CoV-2): Chronik bisheriger Massnahmen und Ergebnisse, https://www.bundesgesundheitsministerium.de/coronavirus/chronik-coronavirus.html Accessed: 10 Dec 2021

[CR37] Handelsblatt GmbH (2021). Corona-Chronik, Die Zusammenfassung der aktuellen Lage seit Ausbruch von COVID-19 im Januar 2020, https://www.handelsblatt.com/politik/corona-chronik-aerztebund-fordert-impfungen-auch-am-wochenende/25584942.html?ticket=ST-5954227-YwTygbsV33Sg76YFxI5k-cas01.example.org Accessed: 10 Dec 2021

[CR38] Gottlieb C, Grobovsek J, Poschke M. (2020) Working from home across countries

[CR39] Green N, Tappin D, Bentley T (2020) Working from home before, during and after the Covid-19 pandemic: Implications for workers and organisations. New Zealand J Emplo Relations 45(2):5–16

[CR40] Hatayama M, Viollaz M, Winkler H. (2020) Jobs’ amenability to working from home: evidence from skills surveys for 53 countries. World Bank Policy Research Working Paper No. 9241

[CR41] International Labor Organization (2020) Working from Home: Estimating the worldwide potential. ILO Policy Brief, https://www.ilo.org/wcmsp5/groups/public/---ed_protect/---protrav/---travail/documents/briefingnote/wcms_743447.pdf Accessed: 10 Feb 2022

[CR42] Ipsen C, van Veldhoven M, Kirchner K, Hansen JP (2021) Six key advantages and disadvantages of working from home in Europe during COVID-19. Int J Environ Res Public Health 18(4):1826 33668505 10.3390/ijerph18041826 PMC7917590

[CR43] JP Morgan Chase & Co (2020). 2020 Annual Report, https://www.jpmorganchase.com/content/dam/jpmc/jpmorgan-chase-and-co/investor-relations/documents/annualreport-2020.pdf Accessed: 31 Jan 2022

[CR44] Kirchner K, Ipsen C, Hansen JP (2021) COVID-19 leadership challenges in knowledge work. Knowl Manag Res Pract 19(4):493–500

[CR45] Milasi S, Gonzales-Vazquez I, Fernandez-Macias E. (2020) Telework in the EU before and after the COVID-19: where we were, where we head to. Science for Policy Briefs European Commission, https://joint-research-centre.ec.europa.eu/system/files/2021-06/jrc120945_policy_brief_-_covid_and_telework_final.pdf.

[CR46] Mongey S, Pilossoph L, Weinberg A. (2020) Which workers bear the burden of social distancing?. National Bureau of Economic Research (NBER). Working Paper No. 27085 10.1007/s10888-021-09487-6 PMC8328128 34366750

[CR47] Nilles J (1975) Telecommunications and organizational decentralization. IEEE Trans Commun 23(10):1142–1147

[CR48] Pranggono B, Arabo A (2021) COVID-19 pandemic cybersecurity issues. Internet Technol Lett 4(2):e247

[CR49] Putri AV, Irwansyah I (2020) Communication patterns and media technology role in organization and society during pandemic. J Soc Media 4(2):228–261

[CR50] Rodeghero P, Zimmermann T, Houck B, Ford D. (2021) Please turn your cameras on: Remote onboarding of software developers during a pandemic. 2021 IEEE/ACM 43rd International Conference on Software Engineering: Software Engineering in Practice (ICSE-SEIP), 41–50

[CR51] Rupietta K, Beckmann M (2018) Working from home. Schmalenbach Bus Rev 70(1):25–55

[CR52] Sahni J (2020) Impact of COVID-19 on employee behavior: Stress and coping mechanism during WFH (Work From Home) among service industry employees. Int J Oper Management 1(1):35–48

[CR53] Thelen K (2019) Transitions to the knowledge economy in Germany, Sweden, and the Netherlands. Comp Polit 51(2):295–315

[CR54] Deutsche W. (2021). Chronologie: Ausbreitung des Coronavirus in Deutschland, https://www.dw.com/de/chronologie-ausbreitung-des-coronavirus-in-deutschland/a-58003172 Accessed: 10 Dec 2021

[CR55] Williams CM, Chaturvedi R, Chakravarthy K (2020) Cybersecurity risks in a pandemic. J Med Internet Res 22(9):e23692 32897869 10.2196/23692 PMC7528623

[CR56] Yang L, Holtz D, Jaffe S, Suri S, Sinha S, Weston J, Joyce C, Shah N, Sherman K, Hecht B, Teevan J (2021) The effects of remote work on collaboration among information workers. Nat Hum Behav 6:43–54 34504299 10.1038/s41562-021-01196-4

